# miR-146a facilitates osteoarthritis by regulating cartilage homeostasis via targeting Camk2d and Ppp3r2

**DOI:** 10.1038/cddis.2017.146

**Published:** 2017-04-06

**Authors:** Xudong Zhang, Chuandong Wang, Jingyu Zhao, Jiajia Xu, Yiyun Geng, Liming Dai, Yan Huang, Sai-Chuen Fu, Kerong Dai, Xiaoling Zhang

**Affiliations:** 1 The Key Laboratory of Stem Cell Biology, Institute of Health Sciences, Shanghai Institutes for Biological Sciences, Chinese Academy of Sciences, Shanghai, China; 2 Department of Orthopedic Surgery, Xin Hua Hospital Affiliated to Shanghai Jiao Tong University School of Medicine, Shanghai, China; 3 Department of Orthopaedics and Traumatology, Faculty of Medicine, The Chinese University of Hong Kong, Hong kong, China

## Abstract

Osteoarthritis (OA), characterized by insufficient extracellular matrix synthesis and cartilage degeneration, is known as an incurable disease because its pathogenesis is poorly elucidated. Thus far, limited information is available regarding the pathophysiological role of microRNAs (miRNAs) in OA. In this study, we investigated the specific function of miR-146a in OA pathophysiology using mouse OA models. We found that the articular cartilage degeneration of miR-146a knockout (KO) mice was alleviated compared with that of the wild-type (WT) mice in spontaneous and instability-induced OA models. We demonstrate that miR-146a aggravated pro-inflammatory cytokines induced suppressing the expression of cartilage matrix-associated genes. We further identified calcium/calmodulin-dependent protein kinase II delta (Camk2d) and protein phosphatase 3, regulatory subunit B, beta isoform (Ppp3r2, also known as calcineurin B, type II) were essential targets of miR-146a in regulating cartilage homeostasis. Moreover, we found that surgical-induced OA mice treated with a miR-146a inhibitor significantly alleviated the destruction of articular cartilage via targeting Camk2d and Ppp3r2. These results suggested that miR-146a has a crucial role in maintaining cartilage homeostasis. MiR-146a inhibition in chondrocytes can be a potential therapeutic strategy to ameliorate OA.

Osteoarthritis (OA) is the most prevalent musculoskeletal disease in the elderly, and it is predicted to affect 60 million people in the United States by 2020.^1, 2^ However, effective disease-modifying therapies for OA are unavailable because of the limited understanding of the disease pathogenesis; as such, joint replacement remains the preferred treatment for patients with advanced OA.^3^

OA is primarily characterized by degradation of the articular cartilage, as well as subchondral bone sclerosis, and osteophyte formation.^4, 5^ It is suggested that OA is caused by the disruption of cartilage homeostatic balance between anabolic and catabolic signals.^6, 7^ Various risk factors, such as abnormal mechanics, aging and inflammation, have been identified to contribute to cartilage destruction.^8, 9, 10^ A better understanding of the underlying molecular mechanisms of deranged cartilage homeostasis may help to develop new treatments for OA.

Recently, the function of microRNAs (miRNAs) in cartilage homeostasis and OA disease received much attention. miRNAs are a class of non-coding small RNAs, which regulate gene expression through mRNA cleavage or translational repression.^11^ Mice with chondrocyte-specific deletion of dicer, which is required for miRNA biogenesis, exhibit severe skeletal growth defects.^12^ This result suggests that miRNAs have a critical role in skeletal development. Several miRNAs have been identified involved in the regulation of cartilage homeostasis. In IL-1*β*-stimulated OA chondrocytes, 42 miRNA were downregulated, 2 miRNA (miR-491 and miR-146a) were upregulated.^13^ MiR-140 was shown greatly reduced in human OA cartilage and is suppressed by IL-1*β* treatment.^14^ Importantly, A disintegrin and metallopeptidase with thrombospondin type 1 motif 5 (*Adamts5*) was identified as a direct target of miR-140, and miR-140 knockout (KO) mice develop more severe OA-like changes compared with wild-type (WT) mice.^15^ Matrix metallopeptidase 13 (MMP13) was identified as a direct target of miR-27b, which is a downregulated miRNA in response to IL-1 stimulation.^13^ It seems that downregulation of these miRNAs is responsible for the cartilage degradation under the effects of IL-1. MiR-146a is intensely expressed in cartilage in early OA, and it is strongly upregulated by IL-1*β* stimulation in cultured normal human articular cartilage chondrocytes.^16^ It has been suggested that miR-146a functions as a negative feedback regulator of pro-inflammatory signaling pathways by targeting TNF receptor-associated factor 6 (*TRAF6*) and interleukin-1 receptor-associated kinase 1 (*IRAK1*) in the THP-1 human monocytic cell line.^17^ However, the exact role of miR-146a in the pathogenesis of OA remains unknown. Other recent findings suggest that miR-146a may contribute to OA pathogenesis by impairing the TGF-*β* signaling pathway via targeting Smad4 and increasing apoptosis.^18, 19^ Further *in vivo* functional analysis is necessary to support this hypothesis. In this study, we sought to determine the roles of miR-146a on cartilage homeostasis and OA development. MiR-146a KO mice and genetic background-matched WT mice were used to develop knee OA with or without induced knee instability. The molecular targets of miR-146a and potential pathway involved in the pathogenesis of OA were further investigated.

## Results

### MiR-146a KO in mice suppresses spontaneous OA

**Figure 1:**
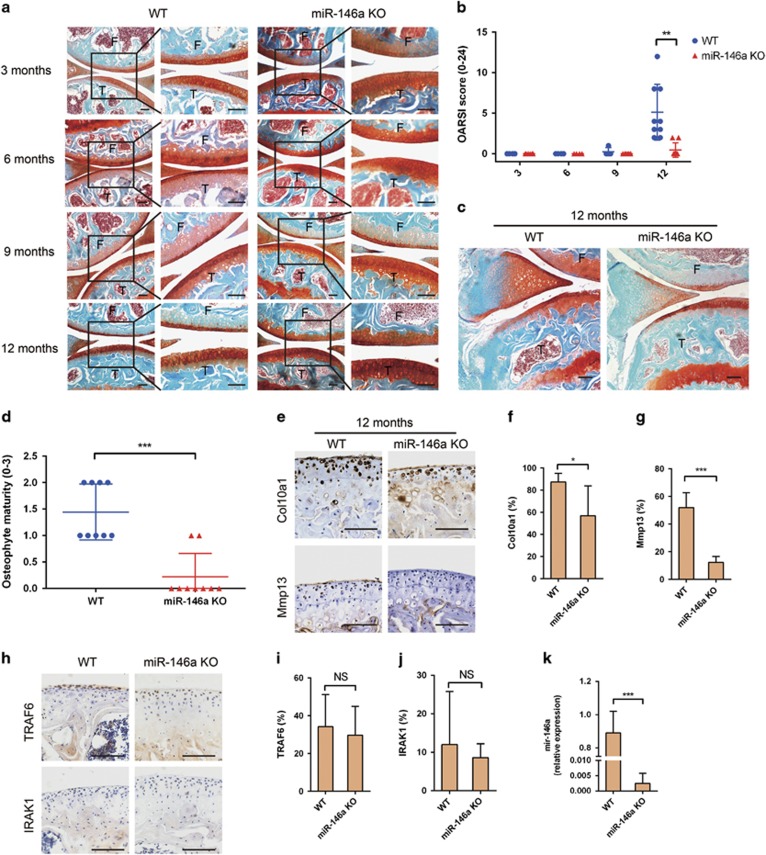
Genetic deletion of miR-146a suppresses spontaneous OA. (**a** and **b**) Safranin O and fast green staining (**a**) or OARSI scores (**b**) of knee joint cartilage harvested from 3-month-old mice (WT, *n*=8; miR-146a KO, *n*=8), 6-month-old mice (WT, *n*=4; miR-146a KO, *n*=9), 9-month-old mice (WT, *n*=4; miR-146a KO, *n*=8) and 12-month-old mice (WT, *n*=9; miR-146a KO, *n*=9). Cartilage (red), bone (blue); F, femur; T, tibia. Scale bars, 100 *μ*m. (**c** and **d**) Safranin O staining and quantification of osteophyte formation in the medial tibial plateau of 12-month-old WT and miR-146a KO mice (WT, *n*=9; miR-146a KO, *n*=9). Scale bars, 100 *μ*m. (**e-g**) Immunohistochemical staining and quantitative analysis of the percentage of Col10a1 and Mmp13-positive chondrocytes (both stained brown) in articular cartilage harvested from 12-month-old mice (WT, *n*=9; miR-146a KO, *n*=9) as described in Materials and Methods section. Sections were counterstained with hematoxylin, nucleus (blue). Scale bars, 100 *μ*m. (**h-j**) Immunohistochemical staining and quantitative analysis of IRAK1 and TRAF6 in knee joint cartilage harvested from 12-month-old mice (WT, *n*=9; miR-146a KO, *n*=9). (**k**) qPCR analysis of miR-146a levels in articular cartilage of 3-month-old mice. (WT, *n*=4; miR-146a KO, *n*=4). Data are mean±S.D. *P<0.05, **P<0.01, ***P<0.001 *versus* WT group (Mann-Whitney test) in **b**, **d**, **f**, **g**, **i**, **j** and **k**. NS, not significant between the indicated groups

At ages of 3, 6 and 9 months, WT and miR-146a KO mice had intact articular cartilage surfaces and vigorous proteoglycan staining in knee joint (Figure 1a). At 12 months of age, the WT mice showed roughened articular surface and glycosaminoglycan loss in femoral condyles and tibial plateaus. In contrast, osteoarthritic changes were less severe in the age-matched miR-146a KO mice, as evident by a higher safranin O staining (Figure 1a). Osteoarthritis Research Society International (OARSI) scores were markedly decreased in miR-146a KO mice compared with that of WT mice, indicating alleviated cartilage degeneration in miR-146a KO mice (Figure 1b). The 12-month-old miR-146a KO mice exhibited reduced osteophyte formation relative to the WT mice (Figures 1c and d). Moreover, the percentages of MMP13 and type X collagen (Col10a1)-positive chondrocytes were markedly lower in 12-month-old KO mice than the age-matched WT mice (Figures 1e and g). The expression of TRAF6 and IRAK1, two well-established targets of miR-146a, did not differ in articular chondrocytes at 12 months between WT and KO mice (Figures 1h and j), suggesting that inflammation may not be under the regulation of miR-146a in spontaneous OA. The absence of miR-146a expression in miR-146a KO mice was confirmed in articular cartilage by qPCR (Figure 1k). These results indicated that deficiency in miR-146a alleviated cartilage degeneration in spontaneous OA model.

### MiR-146a KO in mice alleviates knee destabilization-induced OA

**Figure 2:**
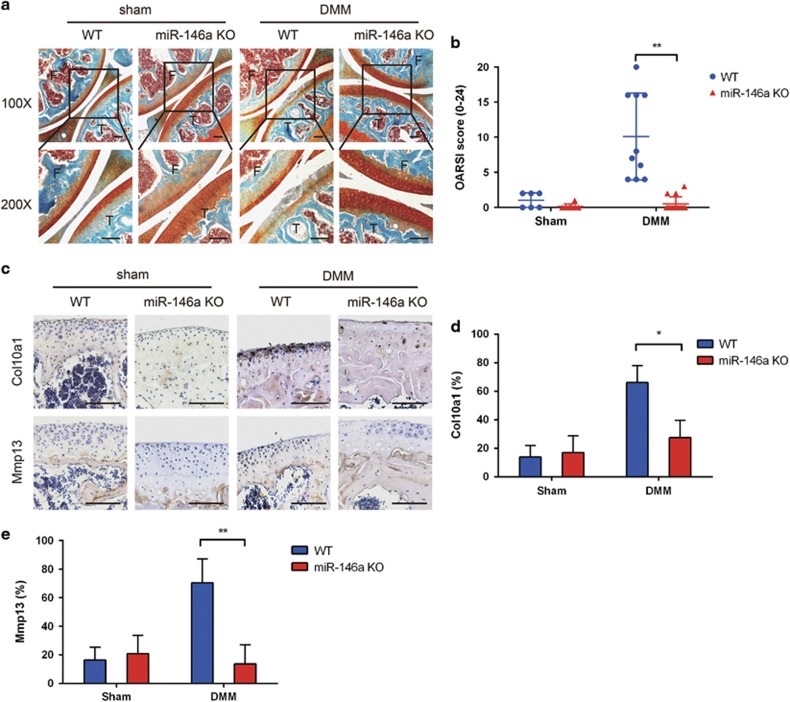
Genetic deletion of miR-146a alleviates cartilage destruction in instability-induced OA models. (**a** and **b**) Safranin O and fast green staining (**a**) or OARSI scores (**b**) of knee joint cartilage sections in WT and miR-146a KO mice collected at 4 weeks after DMM (WT, *n*=10; miR-146a KO, *n*=14) or sham surgery (WT, *n*=6; miR-146a KO, *n*=8). F, femur; T, tibia. Scale bars, 100 *μ*m. (**c-e**) Immunohistochemical staining and quantification of the percentage of Col10a1 and Mmp13-positive chondrocytes in articular cartilage of sham-operated mice (WT, *n*=6; miR-146a KO, *n*=8) and DMM mice (WT, *n*=10; miR-146a KO, *n*=14). Scale bars, 100 *μ*m.Data are mean±S.D. *P<0.05, **P<0.01 (Mann–Whitney test) in **b**, **d** and **e**. NS, not significant between the indicated groups

We used three different knees destabilization-induced OA animal models, namely, destabilization of the medial meniscus (DMM), anterior cruciate ligament transection (ACLT), and medial collateral ligament+ partial medial meniscectomy (PMM), to further evaluate whether the loss of miR-146a affected OA progression. WT mice showed severe proteoglycan loss and articular cartilage degeneration in knee joints at 4 weeks post DMM surgery, but the osteoarthritic changes were less severe in miR-146a KO mice knee joints (Figure 2a). The OARSI scores were not significantly different in sham surgery groups in both KO and WT mice, but markedly decreased OARSI scores in miR-146a KO mice with knee destabilization were observed as compared with the WT mice (Figure 2b). Consistent with this finding, immunostaining demonstrated that the protein expression of type X collagen and Mmp13 was significantly reduced in the miR-146a KO mice compared with that in the WT mice. This result indicated the cartilage degradation may be suppressed (Figures 2c and e). Similar results were observed in ACLT and PMM models (Supplementary Figure 1). These results collectively indicated that the deficiency of miR-146a in mice inhibits the degeneration of articular cartilage during secondary OA development.

### MiR-146a exacerbates pro-inflammatory factors induced suppressing the expression of cartilage matrix-associated genes

**Figure 3:**
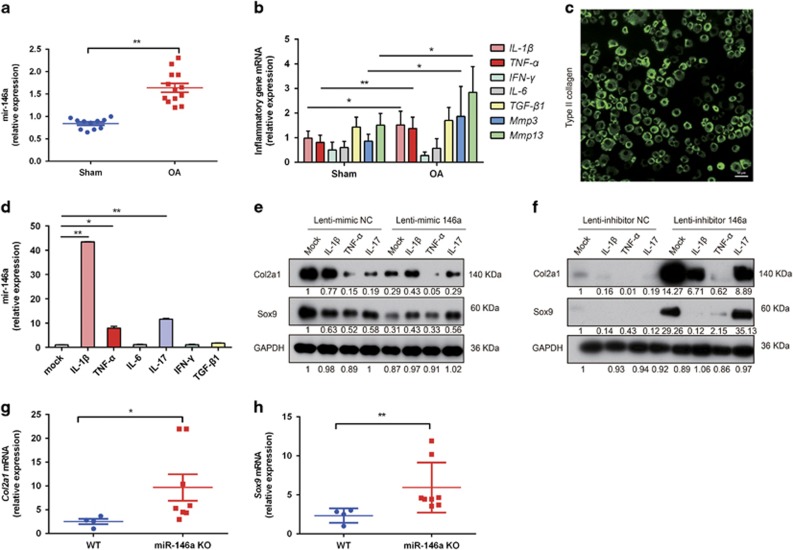
MiR-146a inhibits the expression of cartilage matrix-associated genes. (**a**) qPCR analysis of miR-146a levels in knee joint cartilage of C57BL/6 male mice receiving OA surgery, or sham operation at the age of 12 weeks. Samples were collected at 4 weeks after surgery. Sham, *n*=11; OA, *n*=13. (**b**) qPCR analysis of pro-inflammatory cytokines and MMPs mRNA expression in articular cartilage pooled from 6 sham-operated C57BL/6 mice or 13 mice receiving OA surgery. (**c**) Immunostaining of mouse primary articular chondrocytes with an antibody against type II collagen. Scale bar, 50 *μ*m. (**d**) qPCR analysis of miR-146a levels in mouse primary articular chondrocytes stimulated with IL-1*β* (10 ng/ml), TNF-*α* (10 ng/ml), IL-6 (10 ng/ml), IL-17 (50 ng/ml), IFN-γ (10 ng/ml) or TGF-*β* (10 ng/ml) for 24 h (*n*=3 for each treatment). *P<0.05, **P<0.01 *versus* mock. (**e** and **f**) Mouse primary chondrocytes were infected with Lenti-mimic NC and Lenti-mimic-146a (**e**), or Lenti-inhibitor NC and Lenti-inhibitor 146a (**f**) at 20 MOI for 24 h, then incubated with IL-1*β*, TNF-*α*, IL-17 or left untreated for an additional 48 h (*n*=3 for each treatment). The protein amounts of Col2a1 and Sox9 were determined by western blot. For quantification, protein expression was normalized by the protein amount in the first lane using Image J 1.42 software (NIH, Bethesda, MD, USA). Data are representative of three independent experiments in **d-f**. (**f** and **g**) qPCR analysis of mRNA expression of Sox9 and Col2a1 in knee joint cartilage pooled from four WT mice and eight miR-146a KO mice harvested at 4 weeks after DMM surgery. The relative mRNA expression levels were normalized to the expression of GAPDH. Data are the mean±S.D. *P<0.05, **P<0.01 *versus* control group (Student's *t*-test) in (**d**) and Mann–Whitney test in **a**, **b**, **g** and **h**

We speculated that OA inflammatory microenvironment may be responsible for the miR-146a upregulation. We found that miR-146a was significantly upregulated in the mouse articular cartilage in DMM group as compared with the sham-operated group at 4 weeks post operation (Figure 3a), and higher levels of TNF-*α* and IL-1*β* were also found in DMM groups (Figure 3b). In order to test the effects of pro-inflammatory cytokines on the expression of miR-146a, mouse articular chondrocytes were isolated from the hips and knees of 7-day-old C57BL/6 mice and confirmed by type II collagen expression using immunofluorescence (Figure 3c). We found that IL-1*β*, IL-17 and TNF-*α* upregulated the expression of miR-146a in mouse primary chondrocytes, whereas the other inflammatory cytokines had no effect on miR-146a expression (Figure 3d). Ectopic expression of miR-146a via infection of primary mouse articular chondrocytes with Lenti-mimic 146a reduced the protein levels of type II collagen (Col2a1) and sex determining region Y (SRY)-box 9 (Sox9) in the absence or presence of pro-inflammatory factors (Figure 3e). In contrast, knocking down miR-146a with Lenti-inhibitor 146a markedly upregulated the protein expression of Col2a1 and Sox9, and partially rescued the inhibition of both anabolic genes by pro-inflammatory cytokines (Figure 3f). In the mice with knee destabilization, the expression levels of *Sox9* and *Col2a1* were higher in miR-146a KO mice as compared with WT mice (Figures 3g and h). These results collectively showed that miR-146a, which is induced by pro-inflammatory cytokines, exacerbates suppression of cartilage anabolism in the pathogenesis of OA.

### MiR-146a targets multiple genes to inhibit cartilage anabolism

**Figure 4:**
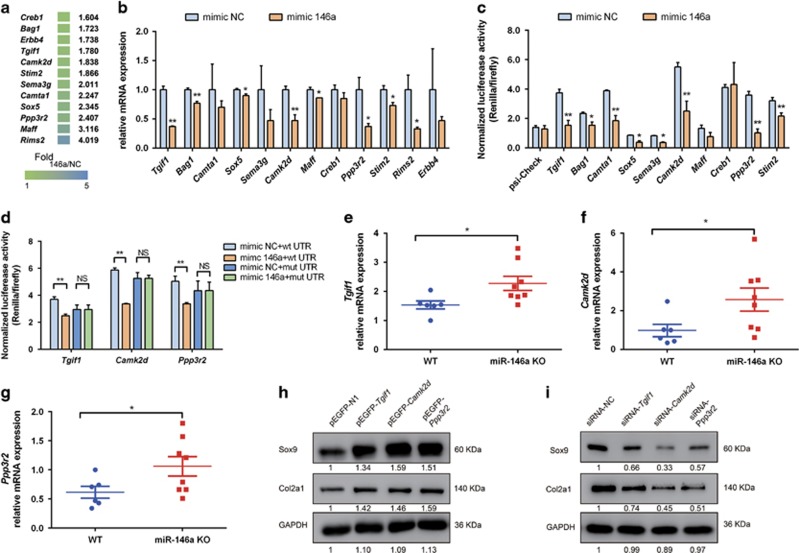
MiR-146a targets Tgif1, Camk2d and Ppp3r2. (**a**) Total RNA of mouse articular chondrocytes transfected with miR-146a inhibitor or control (NC) inhibitor was used for microarray analysis. Twelve genes were validated based on the seed region of miR-146a in 3′-UTR of candidate gene that is evolutionarily conserved in mammals. The gradient colors and numbers indicate fold change of gene expression in mouse chondrocytes transfected with the miR-146a inhibitor compared with control, as determined by microarray. Fold _146a/NC_, fold change of gene expression in mouse chondrocytes transfected with miR-146a inhibitor compared with control inhibitor. (**b**) qPCR analysis of mRNA expression of candidate target genes in mouse articular chondrocytes transfected with miR-146a mimic or control mimic plasmid (*n*=3 for each treatment). (**c** and **d**) Luciferase activity analysis in HEK293T cells that were co-transfected with the indicated 3′-UTR reporter (**c**) or with the indicated WT or mutant 3′-UTR reporter (**d**) along with miR-146a mimic or control mimic plasmid (*n*=3 for each treatment). The results were normalized by internal firefly luciferase readout. (**e-g**) qPCR analysis of mRNA expression of Tgif1 (**e**), Camk2d (**f**) and Ppp3r2 (**g**) in articular cartilage of mice (WT, *n*=6; miR-146a KO, *n*=8) collected at 4 weeks after DMM surgery. (**h** and **i**) Western blot analysis of endogenous Sox9 and Col2a1 protein expression in mouse chondrocytes that were transfected with indicated expression vector constructs (**h**) or with gene-specific siRNAs as well as a scrambled control siRNA (siRNA-NC). For quantification, protein expression was normalized by the protein amount in the first lane. (**i**) Three independent experiments were performed with similar results in **b-d**. Relative mRNA expression was normalized to the expression of GAPDH. Data are mean±S.D. *P<0.05, **P<0.01 *versus* control group (Student's *t*-test) in **b**, **c** and **d** and Mann–Whitney test in **e-g**. NS, not significant between the indicated groups

To explore the molecular mechanisms how miR-146a regulates cartilage homeostasis, we combined target prediction tools such as Miranda, TargetScan and microarray gene expression analyses to search for potential targets of miR-146a. Twelve genes were validated based on the seed region of miR-146a in 3′-untranslated region (UTR) of candidate gene, which is evolutionarily conserved in mammals (Figure 4a). QPCR analysis showed that the expression of the majority of these candidate genes can be suppressed by miR-146a gain-of-function in mouse chondrocytes (Figure 4b). We constructed luciferase reporters containing WT 3′-UTR sequences of candidate genes, which include the miR-146a binding site for the evaluation of direct targeting by miR-146a. The results showed that miR-146a markedly repressed the reporter activity of the 3′-UTR of the genes encoding *Tgif1, Bag1, Camta1, Sox5, Sema3g, Camk2d, Ppp3r2* and *Stim2* (Figure 4c). As the expression of *Tgif1*, *Camk2d* and *Ppp3r2* in cartilage were considerably higher in KO mice as compared with WT mice (both with knee destabilization) (Figures 4e and g), we further investigated if these genes are regulated by direct binding of miR-146a. Mutation of the 3′-UTR of these three genes abolished the regulation by miR-146a (Figure 4d and Supplementary Figure 2A). Moreover, overexpression of *Tgif1,* *Camk2d* and *Ppp3r2* in mouse chondrocytes significantly increased the protein levels of Sox9 and Col2a1 (Figure 4h), which was consistent with the results of inhibition of miR-146a in mouse chondrocytes. Moreover, we found that overexpression of *Tgif1* markedly upregulated *Ppp3r2* levels (Supplementary Figures 2B-D). Knock-down of these three genes by specific siRNA downregulated the expression of Sox9 and Col2a1 (Figure 4i and Supplementary Figure 2E). This result further confirmed that miR-146a could inhibit the expression of cartilage matrix-related genes by targeting *Tgif1, Camk2d* and *Ppp3r2*.

### Regulation of miR-146a level in cartilage has therapeutic effect on OA

**Figure 5:**
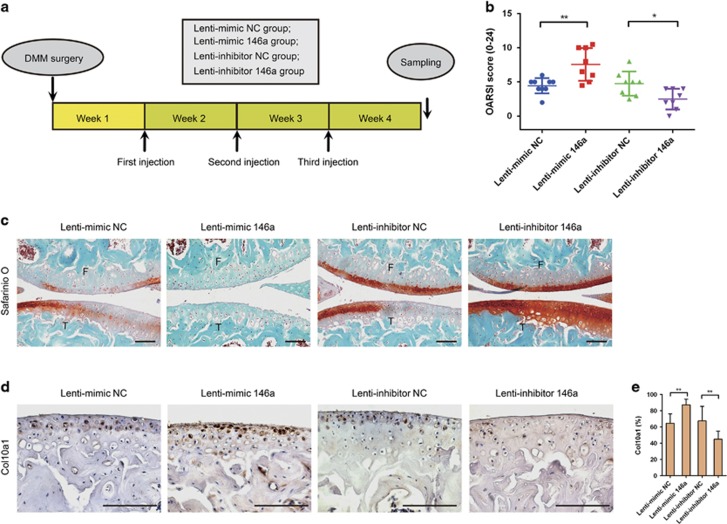
The miR-146a delivery into articular chondrocytes impacted substantially on OA development. (**a**) Schematic diagram illustrating the design of the OA therapeutic experiment. (**b** and **c**) OARSI score (**b**) and Safranin O and fast green staining (**c**) of knee joint cartilage harvested from the indicated groups. F, femur; T, tibia. Scale bars, 100 *μ*m. (**d** and **e**) Immunohistochemical staining and quantitative analysis of the percentage of Col10a1-positive chondrocytes in knee joint cartilage harvested from the indicated groups. *n*=8–9 per group in **a**-**e**. Scale bars, 100 *μ*m. Data are the mean±S.D. *P<0.05, **P<0.01 (Mann–Whitney test)

To investigate whether regulation of miR-146a in OA had a therapeutic effect on OA disease, we performed intra-articular (IA) injection of DMM-operated mice with lentiviruses encoding miR-146a, miR-146a antagonist sequence or corresponding control lentivirus weekly for 3 weeks 1 week after surgery (Figure 5a). The delivery of miRNA to chondrocytes *in vitro* was confirmed by analyzing miR-146a expression in mouse chondrocytes infected with lentiviruses encoding miR-146a, whereas *in vivo* delivery was confirmed by immunostaining of GFP after IA injection of lentivirus integrated with GFP gene (Supplementary Figures 3A and B). We found that DMM mice treated with miR-146a (Lenti-mimic 146) had significantly aggravated the extent of articular cartilage degeneration as compared with the control virus. Conversely, administration of miR-146a inhibitor (Lenti-inhibitor 146a) markedly alleviated articular cartilage degeneration caused by DMM surgery. The therapeutic effect of miR-146a on OA progression was also reflected in OARSI scores (Figures 5b and c). Moreover, the protein expression of Col10a1 was significantly increased by miR-146a overexpression and decreased by miR-146a inhibition (Figures 5d and e), but differences in Mmp13 expression were not significant (Supplementary Figure 3C).

### Camk2d and Ppp3r2 are functional targets of miR-146a *in vivo*

**Figure 6:**
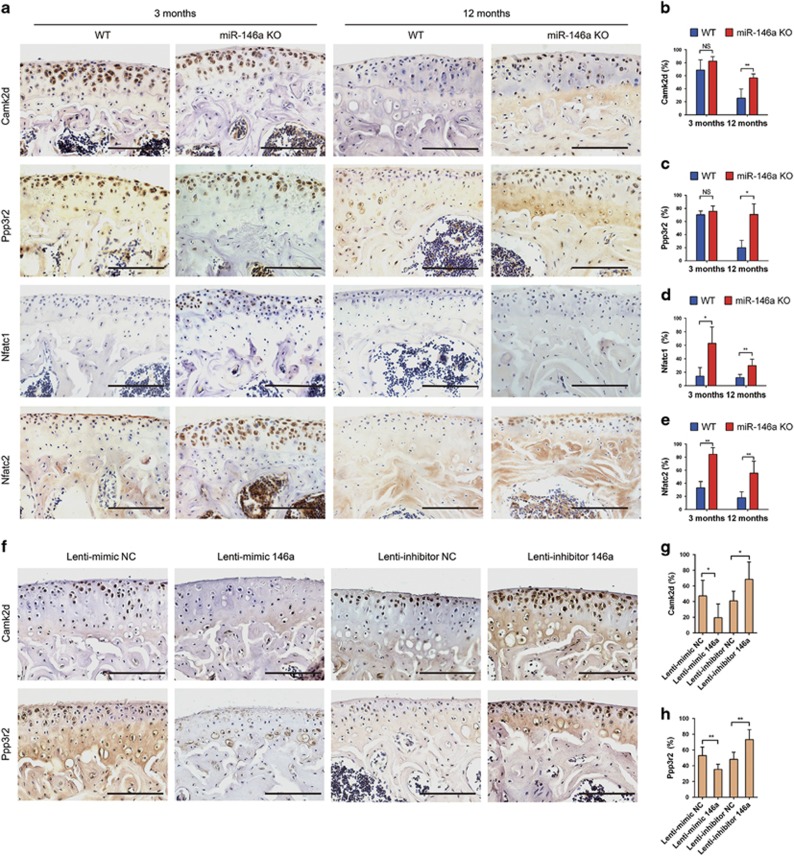
Camk2d and Ppp3r2 are direct targets of miR-146a *in vivo*. (**a-e**) Immunohistochemical staining and quantitative analysis of the percentage of Camk2d, Ppp3r2, Nfatc1 and Nfatc2-positive chondrocytes in knee joint cartilage of 3- and 12-month-old WT or miR-146a KO mice. *n*=8 per group. Scale bars, 100 *μ*m. (**f-h**) Immunohistochemical staining and quantitative analysis of the percentage of Camk2d and Ppp3r2-positive chondrocytes in knee joint cartilage harvested from the indicated groups. *n*=8–9 per group. Scale bars, 100 *μ*m. Data are the mean±S.D. *P<0.05, **P<0.01 (Mann–Whitney test). NS, not significant between the indicated groups

In order to verify the involvement of Tgif1, calcium/calmodulin-dependent protein kinase II delta (Camk2d), and protein phosphatase 3, regulatory subunit B, beta isoform (Ppp3r2) in the pathogenesis of OA, we detected their distribution in mouse knee joints by immunostaining. We found that Camk2d and Ppp3r2 were expressed abundantly at articular cartilage and growth plate in 3-month-old WT mice, but their expression levels were markedly decreased in the articular cartilage after DMM operation (Supplementary Figures 3D and E).The expression of Tgif1 is too low to be detected. The expression levels of Camk2d and Ppp3r2 in articular cartilage were markedly reduced in 12-month-old WT mice compared with that of 3-month-old WT mice. This age-related downregulation of Camk2d and Ppp3r2 was significantly alleviated in KO mice as compared with that in WT mice (Figures 6a and c). We also observed that the expression of Camk2d and Ppp3r2 in bone marrow cells of 3-month-old miR-146a KO mice was much higher than that of age-matched WT mice (Supplementary Figure 3F). The protein expression of Camk2d and Ppp3r2 were downregulated by miR-146a overexpression and upregulated by miR-146a inhibition (Figures 6f and h), it indicated that they are the direct targets of miR-146a *in vivo,* as their 3′-UTR sequences containing miR-146a-binding site. As one of the regulatory subunit of calcineurin, Ppp3r2 is required for the activation of nuclear factor of activated T cells (NFAT) proteins,^20^ which were expressed significantly higher at the hyaline cartilage and bone marrow cells of miR-146a KO mice as compared with age-matched WT mice. The ratio of nuclear NFAT-positive chondrocytes/total NFAT-positive chondrocytes at cartilage was also markedly higher in 12-month-old miR-146a KO mice than age-matched WT mice (Figures 6a, d and e and Supplementary Figure 3F and G). Additional data showed that SMAD family member 4 (SMAD4), a well-known mediator of TGF-*β* pathway, was scarcely distributed at the hyaline cartilage of 12-month-old WT mice, but highly expressed in miR-146a KO mice (Supplementary Figures 3H and I). Collectively, these results suggest that TGF-*β* signaling and calcineurin-mediated activation of NFAT pathway may be involved in the miR-146a regulation of OA progression.

## Discussion

miRNAs may have an important role in the pathogenesis of OA. For the miRNAs downregulated by pro-inflammatory cytokines, previous reports suggest that induction of MMPs and catabolic enzymes by IL-1*β* were regulated by suppression of miR-140, for example, miR-140 targets *Adamts5*, which mediate degeneration of articular cartilage.^14^ However, the function of miRNAs, which are upregulated by pro-inflammatory cytokines treatment in OA remains ambiguous. It is reported that miR-146a, which is induced in response to lipopolysaccharide (LPS) and pro-inflammatory mediator stimulation,^17^ was highly expressed in human RA synovial tissue and less highly expressed in OA tissue.^21^ miR-146a may have the potential to be a novel targets in OA by negative feedback regulation of inflammatory responses,^22, 23, 24, 25^ or by promoting chondrocytes autophagy.^26^ However, other studies showed that miR-146 may contribute to OA pathogenesis by promoting VEGF expression and impairing the TGF-*β* signaling pathway through targeting of Smad4.^18, 19^ In this study, we found that miR-146a was upregulated in cartilage in the early stage of OA induced by knee destabilization (including PMM, ACLT and DMM models). We speculated that OA inflammatory environment may be responsible for the miR-146a upregulation. We found that the mRNA levels of IL-1*β* and TNF-*α* were elevated in OA lesions. IL-1*β* induced highest levels of miR-146a in chondrocytes than that of TNF-*α* and IL-17, indicating that the upregulation of miR-146a in eroded cartilage is correlated with inflammatory cytokines especially IL-1*β* synthesized by resident chondrocytes. It should be noted that mechanical pressure injury and pro-inflammatory cytokines secreted by synovium or meniscus are potentially involved in the induction of miR-146a in OA cartilage.^19, 27^

MiR-146a has been considered as a negative regulator of inflammation,^28^ and administration of miR-146a prevented joint destruction in mice with collagen-induced arthritis.^29^ However, we found that abolishing the expression of miR-146a in mice by removal of miR-146a precursor sequence in mouse genome did not exacerbate the destruction of articular cartilage. On the contrary, KO of miR-146a *in vivo* significantly alleviated the cartilage degeneration of OA caused by aging or imbalanced mechanical loading, suggesting that miR-146a may serve a role other than restriction of inflammation. We found that inhibiting the endogenous expression of miR-146a in mouse primary chondrocytes significantly enhanced the levels of Col2a1 and Sox9, which are well-known markers of mature chondrocytes, and partially rescued pro-inflammatory factors induced inhibition of both anabolic genes. Pro-inflammatory cytokines induced miR-146a strengthened the cytokines-mediated suppression of anabolic genes, suggesting that miR-146a is responsible for the disordered cartilage homeostasis. The role of miR-146a in OA was further confirmed by our *in vivo* therapeutic experiment in which treated DMM mice with miR-146a or miR-146a inhibitor significantly aggravated or alleviated the destruction of articular cartilage, respectively. Our study provides the first insight that regulation of miR-146a level *in vivo* has a therapeutic effect for OA.

We further elucidated the mechanism of miR-146a suppresses cartilage anabolism. We identified the targets of miR-146a, namely, Tgif1, Camk2d and Ppp3r2. CaMKII is a widely distributed Ser/Thr protein kinase that is encoded by four genes (*α*, *β*, *γ* and *δ*).^30^ As soon as CaMKII was activated by Ca^2+^/calmodulin, CaMKII phosphorylated transcription factor cAMP response element (CRE)-binding protein (CREB), thereby facilitating the activation of downstream genes.^31^ In addition to activating CaMKII, Ca^2+^ also activated the phosphatase activity of calcineurin to dephosphorylate NFAT by binding to the 19-kDa regulatory subunit of calcineurin (calcineurin B).^32^ The activated NFAT then translocated to the nucleus and induced the expression of NFAT target genes.^33^ There are two isoforms of calcineurin B exist in mammals, calcineurin B, Type I and calcineurin B, type II.^34^ The two subunit isoforms are indistinguishable in their binding to bacterially expressed forms of mouse catalytic subunits,^35^ and are required for the activation of all NFAT proteins.^20, 36^ Although the importance of CaMK–CREB, as well as calcineurin–NFAT pathway, in osteoclasts and osteoblasts has been well documented,^37, 38, 39, 40^ the contribution of both pathways in OA remains poorly elucidated. In this study, we identified Camk2d and Ppp3r2 were direct targets of miR-146a in the development of OA. Ectopic expression of Camk2d and Ppp3r2 significantly enhanced the protein amounts of Col2a1 and Sox9. Moreover, the expression of Camk2d and Ppp3r2 in normal cartilage is mainly located at the hyaline cartilage or growth plate, seldom distributed at the calcified cartilage or OA cartilage. This result suggests that Camk2d and Ppp3r2 may be required to maintain the phenotype of mature chondrocytes. Although the specific mechanism by which the Camk2d and Ppp3r2 regulate the expression of Col2a1 or Sox9 remains unclear, the calcineurin–NFAT pathway may be involved in the regulation of cartilage homeostasis. We found that miR-146a KO mice expressed more activated Nfatc1 and NFatc2 than WT mice in articular cartilage, especially in hyaline cartilage zone. The calcineurin–NFAT pathway had a critical role in promoting chondrogenesis through induction of Sox9,^41, 42^ or by activating bone morphogenetic protein expression.^43^ Mice lacking Nfatc2, a member of NFAT transcription factor family, displayed loss of Col2a1 and aggrecan (Acan) with high expression of specific matrix-degrading proteinases in adult articular chondrocytes, all of which resemble human OA.^44^ Further study revealed that Nfatc2 bound to the promoter regions of Acan, Col2a1, Mmp13 and Tnfa genes in articular chondrocytes of aged mice,^45^ suggested that large changes in Sox9 and Col2a1 levels caused by overexpression and inhibition of miR-146a in chondrocytes *in vitro* may attribute to regulation of Nfatc2 by miR-146a. Moreover, cartilage-specific ablation of NFATc1 in NFATc2 KO mice led to aggressive OA, as well as upregulation of *Mmp13.*^46^ It may explain why the KO mice have less OA and lower expression of MMPs than the WT mice.

It should be noted that some limitations are present in this study. For example, safer delivery of miRNA to articular chondrocytes *in vivo* should be sought because we found that a mouse died 1 week later after the last injection in the therapeutic experiment. We speculate the undesirable side-effect of lentivirus is the likely cause. Moreover, the role of Tgif1 in the pathogenesis of OA was partly ignored because we failed to detect it *in vivo*. However, Tgif1 may have the same effect as Camk2d and Ppp3r2, as overexpression of Tgif1 in chondrocytes greatly enhanced the expression of Ppp3r2. Further investigation should be performed to elaborate the role of above-mentioned genes in OA.

**Figure 7:**
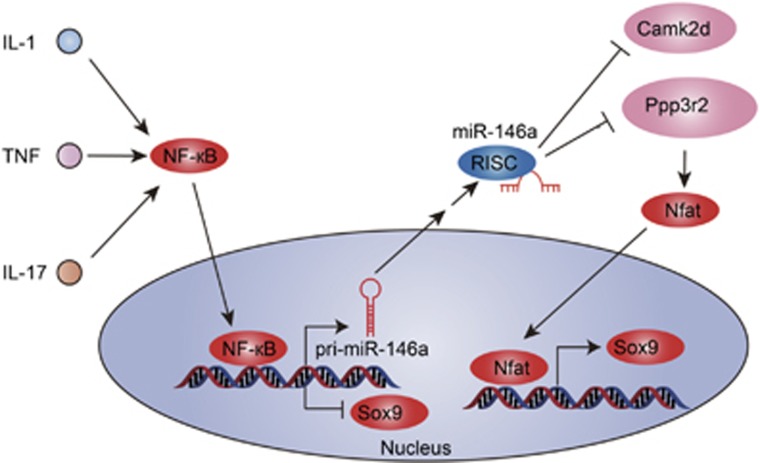
Schematic model of the role of miR-146a in the pathogenesis of OA. MiR-146a is induced in chondrocytes in an NF-кB-dependent manner by pro-inflammatory cytokines stimulation. Once activated, miR-146a disrupts cartilage homeostasis via targeting Camk2d and Ppp3r2, which are associated with cartilage anabolism, thereby inhibiting the activation of downstream NFAT pathway

Inflammatory mediators in joint space greatly reduced the capacity of chondrocytes to repair damaged cartilage by suppressing the cartilage anabolism-related genes. Here, we identified miR-146a, an upregulated miRNA in response to inflammation stimulation, act as an accomplice of pro-inflammatory factors in promoting the progression of OA by targeting Camk2d and Ppp3r2 (Figure 7). Our study provides the first *in vivo* evidence that inhibiting the levels of miR-146 can alleviate cartilage degeneration in OA. MiR-146a may be a potential therapeutic target for OA.

## Materials and methods

### Mice

MiR-146a heterozygous KOs on a C57BL/6 background were obtained from the Jackson Laboratory (Bar Harbor, ME, USA). Upon arrival at our animal facility, the miR-146a heterozygotes were intercrossed. The miR-146a homozygous KOs (designated as miR-146a KO) and WT C57BL/6 mice were screened by genotyping and mating as homozygote × homozygote or WT × WT. The WT and miR-146a KO offspring were used in spontaneous and surgically induced OA models. The primer sequences used for genotyping are as follows: forward primer 5′-ACCAGCAGTCCTCT-TGATGC-3′ and reverse primer 5′-GACGAGCTGCTTCAAGTTCC-3′. C57BL/6J male mice were purchased from Shanghai Laboratory Animal Center (Shanghai, China), Chinese Academy of Sciences (Shanghai, China) and used in OA therapeutic experiment. All mice were maintained under pathogen-free conditions. All animal experiments were approved by the Institutional Biomedical Research Ethics Committee of the Shanghai Institutes for Biological Sciences (Chinese Academy of Sciences).

### Experimental OA

Surgical OA was induced in 12-week-old male miR-146a KO and WT mice through DMM (*n*=10–14 per group), ACLT (*n*=6 per group) or PMM (*n*=6 per group) as previously described.^47, 48, 49^ Briefly, the mice were anesthetized with chloral hydrate (400 mg/kg) intraperitoneally, and the anterior cruciate ligament (for ACLT) or medial meniscotibial ligament (for DMM) was transected in the right knee joint. PMM model was performed by resection of the medial collateral ligament and removal of the cranial horn of the medial meniscus. Sham operation was done on independent mice. Mice were killed 4 weeks (DMM and PMM groups) or 8 weeks (ACLT group) after surgery as previously described,^48, 49, 50^ and right knee joints were processed for histological and biochemical analyses. For spontaneous OA experiments, male mice were killed and right knee joints were harvested from 3-, 6-, 9- and 12-month-old miR-146a KO and WT mice. For the therapeutic experiment, 3-month-old C57BL/6 male mice receiving DMM surgery were assigned into four groups (*n*=8–9 per group). One week after surgery, mice were treated with IA injection of lentivirus-incorporated miR-146a mimic and inhibitor, or corresponding control lentivirus (1 × 10^9^ plaque-forming units in a total volume of 5 *μ*l) once every week for 3 weeks. Mice were killed 3 weeks after the first IA injection for histological analyses.

### Histology and immunohistochemistry

Right knee joints were fixed in 4% paraformaldehyde for 24 h, decalcified in 12.5% EDTA (pH 7.4) for 2 weeks, embedded in paraffin. In all, 5 *μ*m sagittal sections were cut from the whole medial compartment of the joints and stained with safranin O and fast green. Cartilage destruction in the medial tibial plateau of the joint was scored by two blinded observers using the OARSI system.^51^ In short, five serial sections from each individual were scored according to the formula: score=grade × stage. The individual score was calculated by averaging the scores of five sections. Osteophyte development in the medial tibial plateau was determined by safranin O staining and quantified as described previously.^47^ Immunohistochemistry was performed according to the manufacturer's instructions (Maixin Biotech. Co., Ltd, Fuzhou, China). Sections were deparaffinized and rehydrated. Antigen retrieval was performed by digesting with pepsin, trypsin or hyaluronidase (Sigma-Aldrich, St. Louis, MO, USA) at 37 °C for several minutes. Sections were blocked with 5% goat serum, 0.1% tween-20 in phosphate-buffered saline (PBS) for 30 min at room temperature, and then incubated with following primary antibodies to Mmp13 (1:200, ab39012; Abcam, Cambridge, UK), type X collagen (1:1000, ab58632, Abcam), GFP (1:1000, ab290, Abcam), Camk2d (1:100, 20667-1-AP; ProteinTech Group, Chicago, IL, USA), Ppp3r2 (1:100, 14005-1-AP, ProteinTech Group), Nfatc1 (1:50, ab175134, Abcam), Nfatc2 (1:100, 22023-1-AP, ProteinTech Group) and isotype control antibody (ab27478, Abcam) overnight at 4 °C. Streptavidin-horse radish peroxidase (HRP) detection system (Maixin Biotech. Co., Ltd) was used to detect antigen and was visualized with 2, 2′-diaminobenzidine tetrahydrochloride. Sections were then counterstained with hematoxylin, and sealed with mounting medium. Images were obtained with ZEN lite 2011 software and Axio imager A2 microscope (Carl Zeiss, Oberkochen, Germany).

### Cell culture

HEK293T cells were purchased from ATCC (Manassas, VA, USA), and mouse primary articular chondrocytes were isolated from femoral heads, femoral condyles and tibial plateaus of mice postnatal 1 week as previously described.^52^ Chondrocytes were maintained as a monolayer in Dulbecco's modified Eagle's medium (DMEM) supplemented with 10% FBS (Gibco, Thermo Fisher Scientific, Waltham, MA, USA) and 1% penicillin–streptomycin (Hyclone, GE Healthcare Life Sciences, Logan, UT, USA) and identified by type II collagen fluorescence immunoassay. Chondrocytes at passage 1–2 (P1–P2) were used in all experiments.

### Immunofluorescence

Chondrocytes at P1 were seeded in a 24-well culture plate at 2 × 10^4^ cells per well. At 70–80% confluence, the cells were rinsed twice with PBS, fixed in 4% paraformaldehyde for 10 min at room temperature. After three washes in PBS, the cells were blocked with 5% bovine serum albumin (BSA), 0.3% tween-20 in PBS for an hour, and incubated with anti-type II collagen antibody (1 : 200, BS1071; Bioworld Technology, St. Louis Park, MN, USA) diluted in PBS overnight at 4 °C. After three washes in PBS, the cells were incubated with the fluorescein isothiocyanate-labeled secondary antibody diluted 1:1000 in PBS for 2 h at room temperature. After three washes in PBS, images were photographed protected from light using a microscope equipped with a mercury lamp.

### Lentiviruses, plasmids and siRNAs

We purchased lentiviruses that expressed a mature sequence of murine miR-146a (Lenti-mimic 146a) or an antagonist sequence (Lenti-inhibitor 146a) and negative control viruses (GenePharma Co., Ltd, Shanghai, China) to effectively reinforce or silence miR-146a expression *in vitro* and *in vivo*. Chondrocytes were seeded on the 12-well plate 1 day before transduction and then infected with viruses for 24 h. Then, cells were processed for further analyses after 72 h. We also purchased vector-based miR-146a precursor and inhibitor clones (GeneCopoeia, Rockville, MD, USA). For gene expression experiments, full-length mRNA encoding mouse TGFB-induced factor homeobox 1 (*Tgif1*, gene ID 21815), mouse *Camk2d* (gene ID 108058) and mouse*Ppp3r2* (gene ID 19059) were cloned into a pEGFP-N1 vector (Clontech, Takara Bio USA, Inc., Mountain View, CA, USA). siRNAs targeting mouse *Tgif1, Camk2d* and *Ppp3r2* are obtained from GenePharma Co., Ltd. Scrambled siRNA was used as a control. Chondrocytes were transfected with either the expression plasmid or siRNA using Lipofectamine 2000 (Invitrogen, Thermo Fisher Scientific, Waltham, MA, USA). Cells were collected 48 h after transfection for further analyses. Experiments were performed in duplicates and repeated at least three times independently. The sequences of siRNAs and primers that were used for gene cloning can be found in Supplementary Table 1.

### qPCR

Mouse cartilage was cut with a surgical blade from the medial tibial plateau and medial femoral condyle; avoid the contamination from the brown subchondral bone. Cartilage was rinsed with PBS, transferred to 1 ml TRIzol reagent (Invitrogen), and then homogenized with a power homogenizer at highest speed on ice. Total RNA was extracted from homogenized cartilage samples or primary cultured chondrocytes with TRIzol reagent and treated with DNase I (Sigma-Aldrich) to remove genomic DNA. For mRNA expression, RNA was reverse transcribed into complementary DNA using a PrimeScript RT Master Mix Kit (Takara Bio Inc., Dalian, China) and then analyzed using real-time PCR (SYBR Green). All mRNA expression was normalized by housekeeping gene glyceladehyde-3-phosphate dehydrogenase (*GAPDH*). qPCR primers are summarized in Supplementary Table 2. Mature miR-146a were assayed with specific Taqman kits from Applied Biosystems (Thermo Fisher Scientific, Waltham, MA, USA) and normalized by U6 snRNA.

### Western blot analysis

Cells were lysed in lysis buffer (1 × PBS, 0.1% SDS, 0.5% sodium deoxycholate, 1% NP-40, 5 mM EDTA, 1 mM PMSF and protease inhibitor cocktail), subjected to SDS-PAGE, and transferred to a nitrocellulose membrane. The membranes were blocked with 5% BSA and probed with antibodies against COL2A1 (1:1000, BS1071; Bioworld Technology), SOX9 (1:1000, ab26414, Abcam) or GAPDH (1:5000, G9545, Sigma-Aldrich).

### 3′-UTR cloning and luciferase assay

The 3′-UTRs of candidate genes containing the predicted miR-146a target sequences were PCR amplified and then inserted between *Xho*I and *Not*I site downstream from the *Renilla* luciferase gene in a psiCHECK-2 vector (Promega, Sunnyvale, CA, USA). Binding site mutations were performed using QuikChange Lightning Site-Directed Mutagenesis Kit (Agilent Technologies, Inc., Santa Clara, CA, USA). Primers that used for 3′-UTRs cloning are summarized in Supplementary Table 3. For luciferase assay, 293T cells were seeded at 3000 cells per well in a 96-well plate. The cells in each well were transfected with a mixture of 20 ng of luciferase constructs and 80 ng of miR-146a precursor mimic or control mimic plasmid after 24 h. At 48 h after transfection, cells were lysed, and luciferase activity was measured on a luminometer using dual-luciferase reporter assay system (Promega) according to the manufacturer's instructions. Luciferase activity was normalized by *firefly* luciferase activity. Experiments were performed in triplicate and repeated at least three times independently.

### mRNA microarray

Total RNA was isolated from mouse chondrocytes transfected with miR-146a inhibitor or control inhibitor using TRIzol reagent. RNA integrity was assessed using standard denaturing agarose gel electrophoresis. Whole Mouse Genome Oligo Microarray (4x44K, Agilent Technologies) platform was used for microarray analysis. Sample preparation and microarray hybridization were performed based on the manufacturer's standard protocols. Agilent Feature Extraction software (version 11.0.1.1, Agilent Technologies, Inc., Santa Clara, CA, USA) was used to analyze acquired array images. Quantile normalization and subsequent data processing were performed using the GeneSpring GX v11.5.1 software package (Agilent Technologies).

### Statistical analyses

All statistical analyses were performed with SPSS 19.0 software (SPSS Inc., IBM Corporation, Armonk, NY, USA). Data are presented as mean±S.D. Statistical differences between two groups were determined by two-tailed Student's *t-*test or a Mann–Whitney test. *P*<0.05 was considered statistically significant.
