# Supplementary figures and images for: miR-146a facilitates osteoarthritis by regulating cartilage homeostasis via targeting Camk2d and Ppp3r2
Source: Cell Death Dis. 2017 Apr 6;8(4):e2734–. doi: 10.1038/cddis.2017.146 (PMC5477577; doi:10.1038/cddis.2017.146)

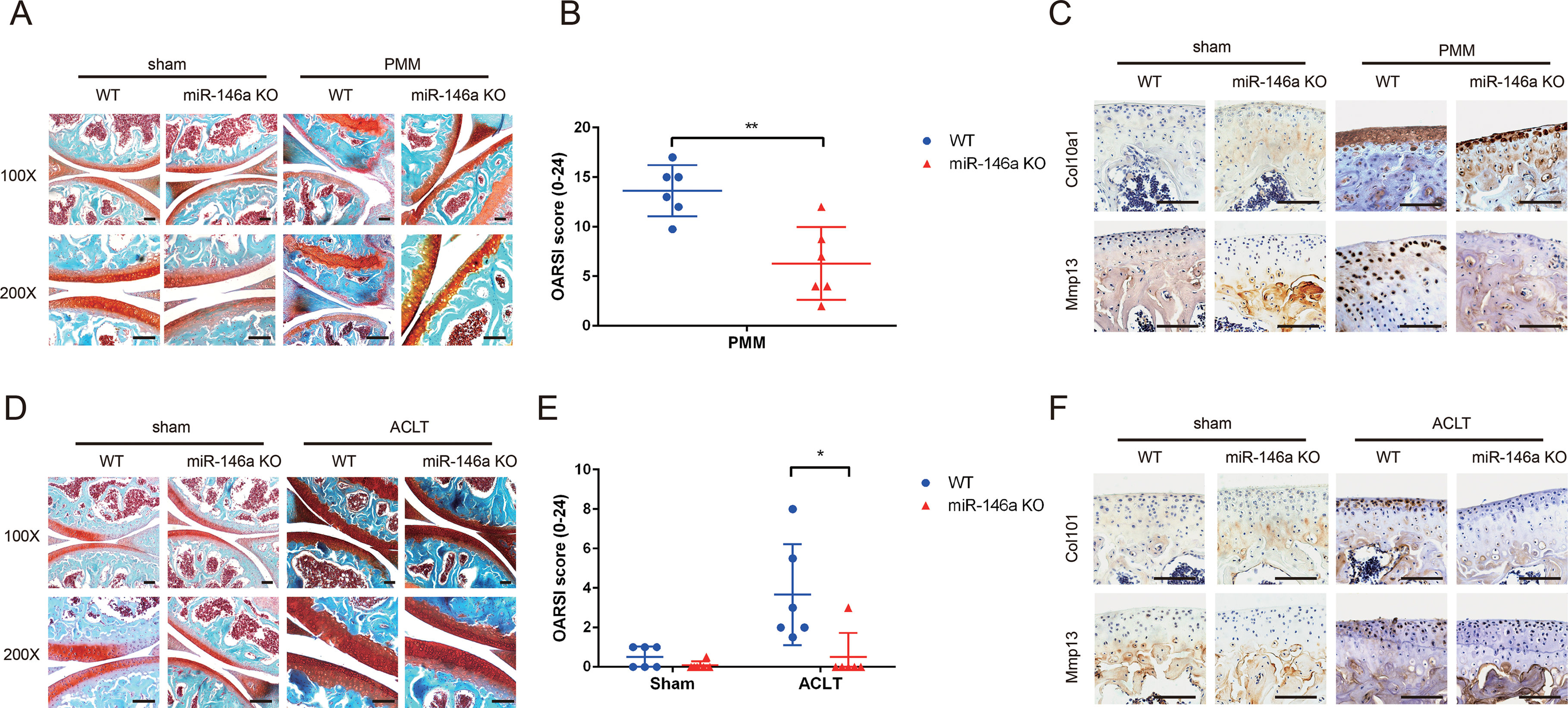

Supplement: Supplementary Figure 1 [file cddis2017146x1.tif]

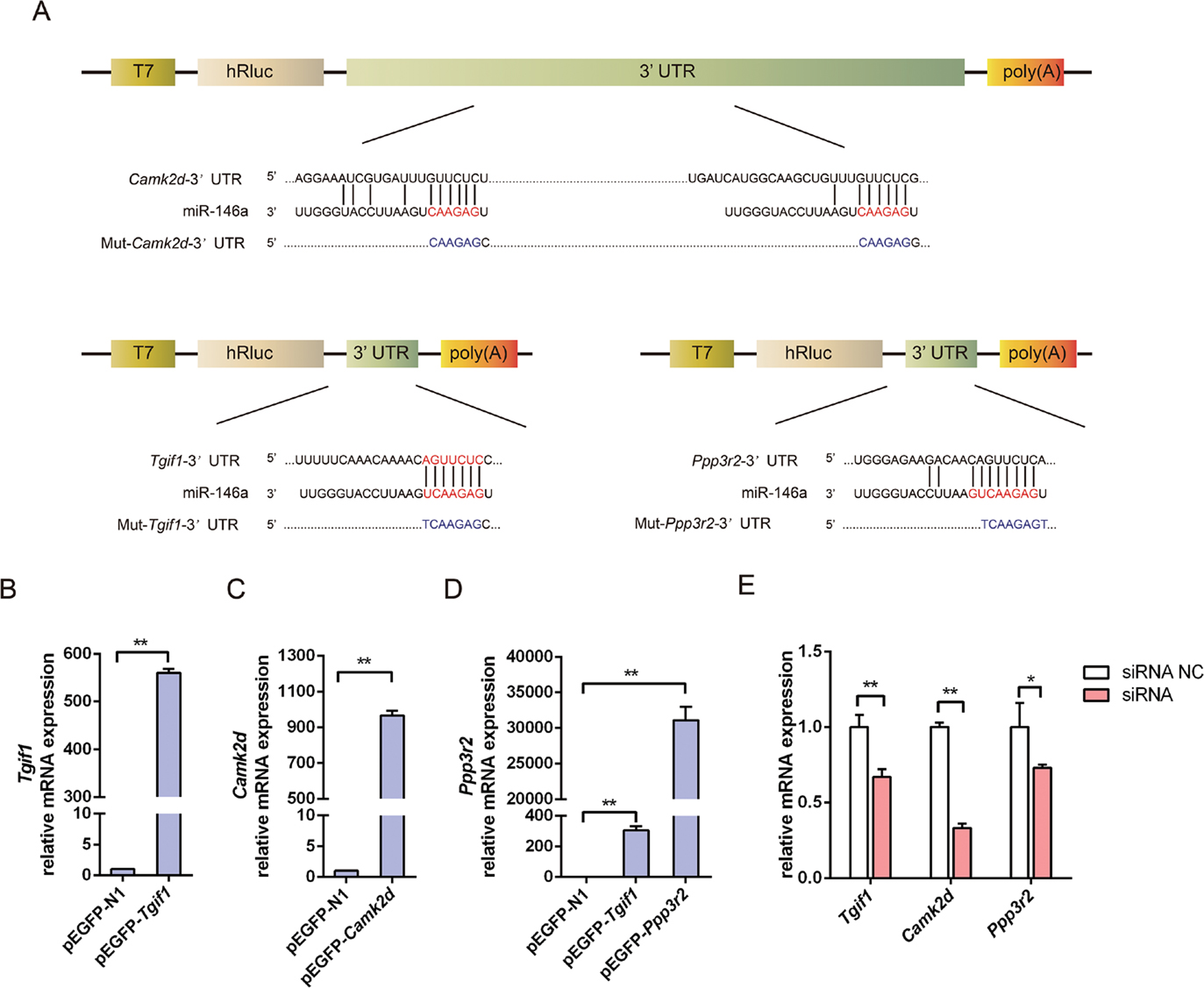

Supplement: Supplementary Figure 2 [file cddis2017146x2.tif]

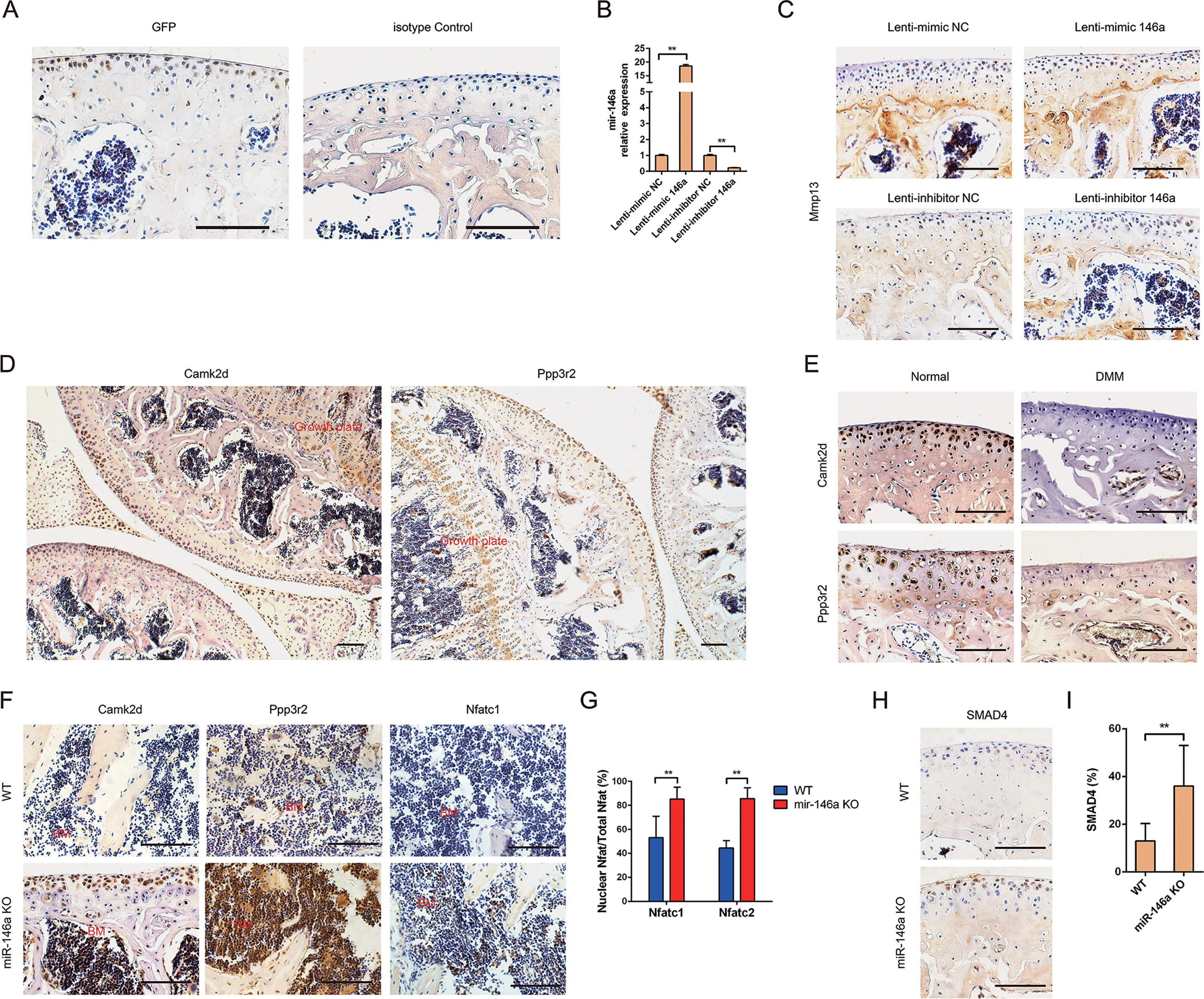

Supplement: Supplementary Figure 3 [file cddis2017146x3.tif]
